# Body Appreciation in Lesbian, Bisexual, and Queer Women: Examining a Model of Social Support, Resilience, and Self-Esteem

**DOI:** 10.1089/heq.2019.0003

**Published:** 2019-05-20

**Authors:** C. Blair Burnette, Melissa A. Kwitowski, Michael A. Trujillo, Paul B. Perrin

**Affiliations:** Department of Psychology, Virginia Commonwealth University, Richmond, Virginia.

**Keywords:** body appreciation, women, sexual minority, social support, resilience, self-esteem

## Abstract

**Purpose:** There is increasing research on positive body image, but no studies to date have examined these constructs in lesbian, bisexual, and queer (LBQ) women. However, LBQ women are at increased risk for mental health concerns and disordered eating, and there is evidence that body appreciation might be both adaptive and protective. This study examined factors that could uniquely relate to body appreciation in LBQ women.

**Method:** Women identifying as LBQ (*N*=150) completed demographics and measures of social support, resilience, self-esteem, and body appreciation. We tested a hypothesized mediational model of social support leading indirectly to body appreciation through resilience and self-esteem, controlling for body mass index.

**Results:** All direct effects, except social support to body appreciation (*p*=0.696), were significant (*p*s=0.017–0.001), reflecting a full multiple mediation. As hypothesized, the effect of social support on body appreciation was indirect (*p*=0.011), through resilience and self-esteem.

**Conclusion:** This is the first study to investigate factors that might facilitate positive body image in LBQ women. Although preliminary, results suggest social support, resilience, and self-esteem might be important targets of body image interventions with LBQ women.

Body dissatisfaction is endemic among women in Western societies, and is related not only to disordered eating and eating disorders (EDs), but also smoking initiation, depression, suicidal ideation, binge eating, reduced fruit and vegetable intake, and sedentary behaviors.^1–6^ The role of sexual identity in body image is understudied and findings to date are mixed. Some studies suggest lesbian women experience less body dissatisfaction,^7–9^ which researchers posit could result from lesbian women being more likely to reject heteronormative beauty ideals and accept diverse body types.^8,10,11^ Conversely, in other studies, no significant difference in body dissatisfaction between lesbian and heterosexual women has been found.^12–14^

One theory for the lack of differences is that all women in Westernized cultures, regardless of sexual identity, are socialized to measure themselves by and conform to rigid beauty ideals that emphasize thinness and femininity.^15^ There is considerably less research on the experiences of women identifying as bisexual, pansexual, or queer.^16–18^ However, extant evidence confirms that body image is multidimensional,^19^ with sexual identity being the only one influential factor. Moreover, there is considerable within-group heterogeneity. For instance, degree of femininity/masculinity/androgyny, peer sexual orientation, partner choice, media representations, and fashion all seem to contribute to body image in lesbian, bisexual, and queer (LBQ) women.^16–18^

There is growing interest in positive body image as a protective factor against body dissatisfaction and its related consequences.^20^ Positive body image is multidimensional, and one facet, body appreciation, refers to an appreciation of one's body that extends beyond appearance.^21^ Body appreciation is related to adaptive media strategies (e.g., protective filtering),^22^ increased self-esteem, and proactive coping strategies, even when controlling for appearance dissatisfaction.^21^ Body appreciation might also reduce the effects of thin-ideal media exposure.^23^ Because of its adaptive nature and potential as a protective factor, it is an increasing target of intervention work.^24,25^ Despite its promise, there is no known research examining body appreciation in LBQ women.

It is unclear if LBQ women experience less body dissatisfaction; however, disordered eating levels seem comparable, or even greater than, heterosexual women.^26,27^ Moreover, LBQ women are exposed to greater prejudice and discrimination, report greater mental health concerns, and report greater unmet mental health needs.^28^ Body image is an important component of mental health that is associated with a spectrum of mental and physical health outcomes.^1^ Thus, examining factors that facilitate positive body image in a group vulnerable to mental health concerns and inadequate mental health treatment is imperative.

The determinants of body image in LBQ women are complex.^18^ Some factors that might be uniquely related to body image in this group include social support, resilience, and self-esteem. The literature on the benefits of positive social support to physical and mental health is robust.^29^ Social support seems particularly important in the LBQ community as it is related to identity development and adjustment, and can buffer the effects of social stress and rejection.^30–32^ Positive social support is an important resource that strengthens resilience, the successful adaptation to challenging circumstances, particularly in the LBQ community.^29,33^ For instance, in a study of LBQ women who experienced family rejection, those with greater resilience had sought out and developed connections with other members of their LBQ communities.^32^ One way social support aids resilience in LBQ individuals is through lowering reactivity to prejudice, consistent with minority stress theory.^34,35^

Both positive social support and resilience are related to self-esteem. For instance, social support through minority community participation appears to promote an increased sense of both individual and collective self-esteem as a result of the affiliation with other minority individuals.^32^ Although research often examines self-esteem as a protective factor for resilience, resilience also seems to foster self-esteem, potentially through positive affect and self-efficacy.^36,37^ Self-esteem is closely tied to body image, particularly in women.^38,39^ There is scant research in LBQ women, but Striegel-Moore et al.^12^ found self-esteem was *more* strongly related to body esteem in lesbian than heterosexual women.

The purpose of this study was to examine factors that facilitate body appreciation in LBQ women. We hypothesized a multiple mediational model, where social support would be indirectly related to body appreciation through resilience and self-esteem (Fig. 1). We expected positive direct associations between social support and both resilience and self-esteem, resilience and both self-esteem and body appreciation, and self-esteem and body appreciation. We did not expect a significant direct association between social support and body appreciation after controlling for this series of mediators. Current body image interventions do not consider the role of sexual identity. This study will address a gap in the literature, and findings could inform the development and refinement of interventions for LBQ women.

**FIG. 1.**
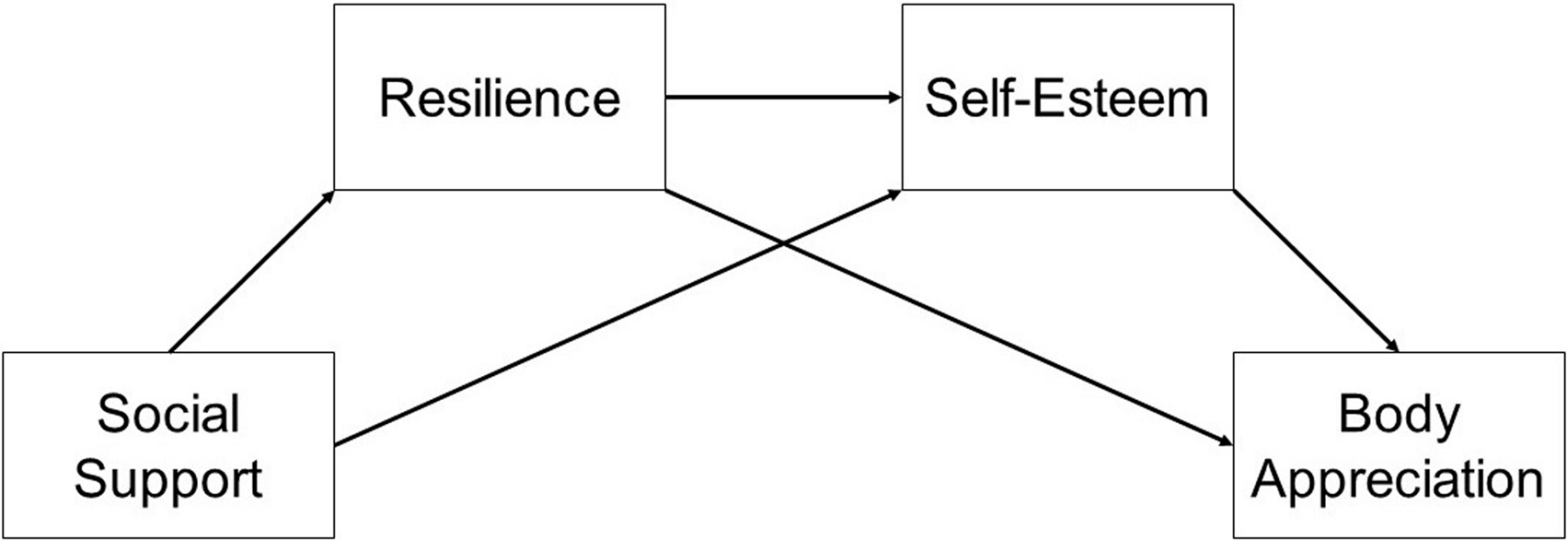
The hypothesized multiple mediational model.

## Method

### Participants and procedure

The host university's Institutional Review Board approved the study. Participants were recruited as part of a larger study on the mental and physical health of lesbian, gay, bisexual, transgender, and queer (LGBTQ) individuals. Recruitment occurred through Internet forums and groups and emails to national (i.e., United States) and regional (i.e., within the United States) LGBTQ organizations. The purpose of this study was described as an investigation of the physical and mental health of racial/ethnic minorities who identify as LGBTQ. Interested individuals emailed the research coordinator who screened for eligibility (i.e., at least 18 years old and identifying as a sexual minority woman). Eligible individuals accessed the online survey through a link and access code sent through email. Electronic consent was obtained. Participants received a $15 gift card upon study completion.

The sample (*N*=150) identified as 32.7% bisexual (*n*=49), 38.7% gay/lesbian (*n*=58), and 28.6% queer/other (*n*=43). Five of the six women identifying as “other” described their sexual orientation as pansexual. Women ranged between 18 and 66 years (*M*=31.90, *SD*=11.95). The sample was fairly racially/ethnically diverse, with 29.3% White (*n*=44), 26.0% Black (*n*=39), 16.7% Asian/Hawaiian/Pacific Islander (*n*=25), 12.7% multiracial (*n*=19), 11.3% Latinx (*n*=17), 2.7% American Indian (*n*=4), and 1.3% “other” (*n*=2).

Six percent of the sample (*n*=9) had received their high school diploma or General Education Development Certificate, 24.7% (*n*=37) had attended some college without a degree, 9.3% (*n*=14) had a 2-year degree, 30.0% (*n*=45) had a 4-year degree, 22.0% (*n*=33) had a master's degree, and 8.0% (*n*=12) had a doctorate. Forty-four percent of the sample (*n*=66) reported being employed full-time, 12.0% (*n*=18) part-time, 18% (*n*=27) were both employed and a student, 16.0% (*n*=24) were students, and 10.0% (*n*=15) were unemployed. The majority of the sample reported being in some type of romantic relationship, with 42.7% (*n*=64) reporting a long-term (>12 months) monogamous relationship, 13.3% (*n*=20) a new (<12 months) monogamous relationship, 14.0% (*n*=21) dating more than one person, and 30.0% (*n*=45) not currently dating or in a relationship. See Table 1 for participant characteristics displayed by sexual orientation and overall.

**Table 1.** Sample Characteristics

	Bisexual (*n*=58)	Lesbian (*n*=49)	Queer (*n*=43)	Overall (*N*=150)
Age (range: 18–66)	31.53 (10.11)	37.09 (13.95)	25.33 (6.65)	31.90 (11.95)
Race/ethnicity				
White/European American	34.7% (17)	31.0% (18)	20.9% (9)	29.3% (44)
Black/African American	22.4% (11)	25.9% (15)	30.2% (13)	26.0% (39)
Asian/Pacific Islander	8.2% (4)	13.8% (8)	30.2% (13)	16.7% (25)
Multiracial	18.4% (9)	10.3% (6)	9.3% (4)	12.7% (19)
Latina	12.2% (6)	13.8% (8)	7.0% (3)	11.3% (17)
American Indian	2.0% (1)	3.4% (2)	2.3% (1)	2.7% (4)
Other	2.0% (1)	1.7% (1)	—	1.3% (2)
BMI	27.34 (6.91)	27.63 (7.95)	30.67 (10.91)	28.41 (8.67)
<18.5	2.0% (1)	6.9% (4)	4.7% (2)	4.7% (7)
18.5–24.9	49.0% (24)	43.1% (25)	34.9% (15)	42.7% (64)
25–29.9	18.4% (9)	17.2% (10)	23.3% (10)	19.3% (29)
≥30	30.6% (15)	32.8% (19)	37.2% (16)	33.3% (50)
SS (range: 12–60)	45.18 (9.16)	44.93 (10.57)	43.77 (10.35)	44.68 (10.01)
BRS (range: 6–30)	18.37 (4.81)	19.12 (5.60)	17.26 (5.07)	18.34 (5.22)
RSES (range: 0–30)	17.94 (5.71)	19.57 (5.60)	16.35 (6.74)	18.11 (6.09)
BAS (range: 1.23–4.92)	3.36 (0.72)	3.52 (0.66)	3.28 (0.90)	3.40 (0.76)

*Note:* Continuous variables are presented as M(SD). Categorical variables are presented as %(*n*).

BAS, Body Appreciation Scale; BMI, body mass index; BRS, Brief Resilience Scale; RSES, Rosenberg Self-Esteem Scale; SS, social support.

### Measures

#### Demographics

Participants were surveyed on their gender, sexual, and racial/ethnic identities, age, height, weight, social class, education level, and employment and relationship status.

#### Body Appreciation Scale

The Body Appreciation Scale (BAS)^40^ is a 13-item measure of an individual's body appreciation, acceptance, and respect. Items are rated from 1=*Never* to 5=*Always* and averaged to derive a total score, with higher scores reflecting greater body appreciation. The BAS was updated after data collection; nevertheless, the original BAS demonstrated good internal consistency, reliability, and construct validity with college women.^41^ Cronbach's alpha in this study was 0.92.

#### Rosenberg Self-Esteem Scale

The Rosenberg Self-Esteem Scale (RSES)^42^ contains 10 items, 5 negatively worded, which measure positive self-evaluations. Items are rated 1=*Strongly Disagree* to 4=*Strongly Agree*. In this study, negatively worded items were reverse coded and items were summed to derive an overall score. Higher scores indicate greater self-esteem. Cronbach's alpha was 0.92.

#### Brief Resilience Scale

The Brief Resilience Scale (BRS)^43^ measures the ability to recover from stress, and is not specific to sexual or gender identity. Six items are rated from 1=*Strongly Disagree* to 5=*Strongly Agree*, with three reverse coded. An example item states, “I tend to bounce back quickly after hard times.” The total score represents the sum of item responses, with higher scores reflecting greater resilience. Cronbach's alpha was 0.88.

#### Multidimensional Scale of Perceived Social Support

The Multidimensional Scale of Perceived Social Support (MSPSS)^44^ assesses the subjective impression of social support adequacy in three specific domains: friends, family, and significant others. Twelve items are rated from 1=*Strongly Disagree* to 5=*Strongly Agree.* Total scores represent the sum of responses, with higher scores indicating greater perceived support. Cronbach's alpha was 0.91.

### Data analysis

#### Preliminary analyses

Participant data were deleted from the survey software if there was an indication of impossible response patterns (e.g., selecting the first response for every single item), responses from a computer (i.e., <20 min or >24 h to complete), or if participants did not correctly respond to four of six (66.6%) randomly placed validation questions (e.g., “Please select strongly agree for the item below”). Assumptions of planned analyses, including normality, and univariate and multivariate outliers, were assessed. Descriptive statistics and bivariate correlations between study variables were conducted using SPSS Version 25.0.^45^ We examined the associations between age, sexual orientation, race/ethnicity, body mass index (BMI), and primary study variables, and significant associations were considered as covariates. One-way between-subjects analyses of variance explored differences on age, BMI, and outcome variables across sexual orientation (i.e., bisexual, lesbian, and queer). Significant omnibus tests were followed by Tukey *post hoc* comparisons to locate differences. We judged significance at α=0.01 to account for multiple comparisons.

#### Path analysis

A mediational path model was developed using AMOS^46^ to examine a hypothesized series of connections leading from social support to body appreciation. See Figure 1 for the hypothesized model. Direct and indirect effects were estimated using 2000 bootstrap samples and bias-corrected 95% confidence intervals. Because the sample size in this study was lower than the 200 recommended by Boomsma and Hoogland,^47^ fit indices were omitted as they would likely be more obscuring than illuminating. Instead, the focus was on direct and indirect effects. However, our sample size exceeded the acceptable ratio of 10 cases for each free parameter,^48^ supporting the appropriateness of path analysis. Because extant research suggests that body image might differ across sexual orientation,^18^ we ran the model overall and separately by sexual orientation. However, because the small group sizes reduced power to detect effects, these analyses were exploratory in nature.

## Results

### Preliminary analyses

Skewness and kurtosis for all variables were normal (coefficients ±1.5). Z-scores were <3.0 *SD*s and visual inspection of scatterplots did not reveal any outliers.^49^ There was a significant negative correlation (*r*=−0.21, *p*=0.011) between BMI and body appreciation; thus, BMI was included as a covariate. See Table 1 for descriptive statistics. There were no significant group differences on study variables, except for age. A *post hoc* Tukey test revealed that the queer sample was significantly younger than the lesbian sample (*p*<0.001).

### Mediational path model

A path model examined direct and indirect effects of social support on body appreciation through resilience and self-esteem, controlling for BMI. See Table 2 for an overview of direct and indirect effects. To control for BMI, a path was drawn from BMI to body appreciation. In the first model, we drew paths from social support to resilience, self-esteem, and body appreciation; from resilience to self-esteem and body appreciation; and from self-esteem to body appreciation. As hypothesized, the path from social support to body appreciation was not significant (β=−0.022, *SE*=0.071, *p*=0.696). We trimmed the nonsignificant path and re-ran the model. Because direct and indirect effect estimates remained largely unchanged, we present results only for the final model.

**Table 2.** Bootstrapped, Standardized Estimates of the Multiple Mediational Path Analysis

	Bisexual (*n*=49)		Lesbian (*n*=58)		Queer (*n*=43)		Total sample (*N*=150)	
	β (*SE*)	*p*	β (*SE*)	*p*	β (*SE*)	*p*	β (*SE*)	*p*
Direct effects								
BMI→BAS	**−0.340** (0.104)	0.002	−0.140 (0.091)	0.149	−0.197 (0.113)	0.065	−**0.226** (0.061)	0.002
SS→BRS	**0.292** (0.109)	0.018	**0**.**509** (0.108)	0.001	0.266 (0.153)	0.097	**0.382** (0.074)	0.001
SS→RSES	0.291 (0.148)	0.084	0.206 (0.113)	0.104	**0**.**319** (0.121)	0.017	**0.268** (0.074)	0.002
BRS→RSES	0.277 (0.137)	0.077	0.**571** (0.092)	0.001	**0**.**445** (0.119)	0.005	**0**.**445** (0.069)	0.001
BRS→BAS	**0.345** (0.117)	0.008	−0.083 (0.131)	0.541	0.138 (0.130)	0.240	**0**.**180** (0.076)	0.017
RSES→BAS	0.176 (0.150)	0.305	**0**.**683** (0.106)	0.001	**0**.**579** (0.110)	0.002	**0**.**458** (0.082)	0.001
Indirect effects								
SS→RSES	**0.081** (0.055)	0.044	**0**.**291** (0.081)	<0.001	0.118 (0.080)	0.059	**0**.**170** (0.043)	0.001
BRS→BAS	0.049 (0.056)	0.222	**0**.**390** (0.097)	0.001	**0**.**258** (0.092)	0.004	**0**.**204** (0.054)	0.001
SS→BAS	**0.166** (0.079)	0.018	**0**.**297** (0.090)	0.001	**0**.**290** (0.095)	0.004	**0**.**269** (0.053)	0.011

Parameters significant at *p*<0.05 are given in bold.

**FIG. 2.**
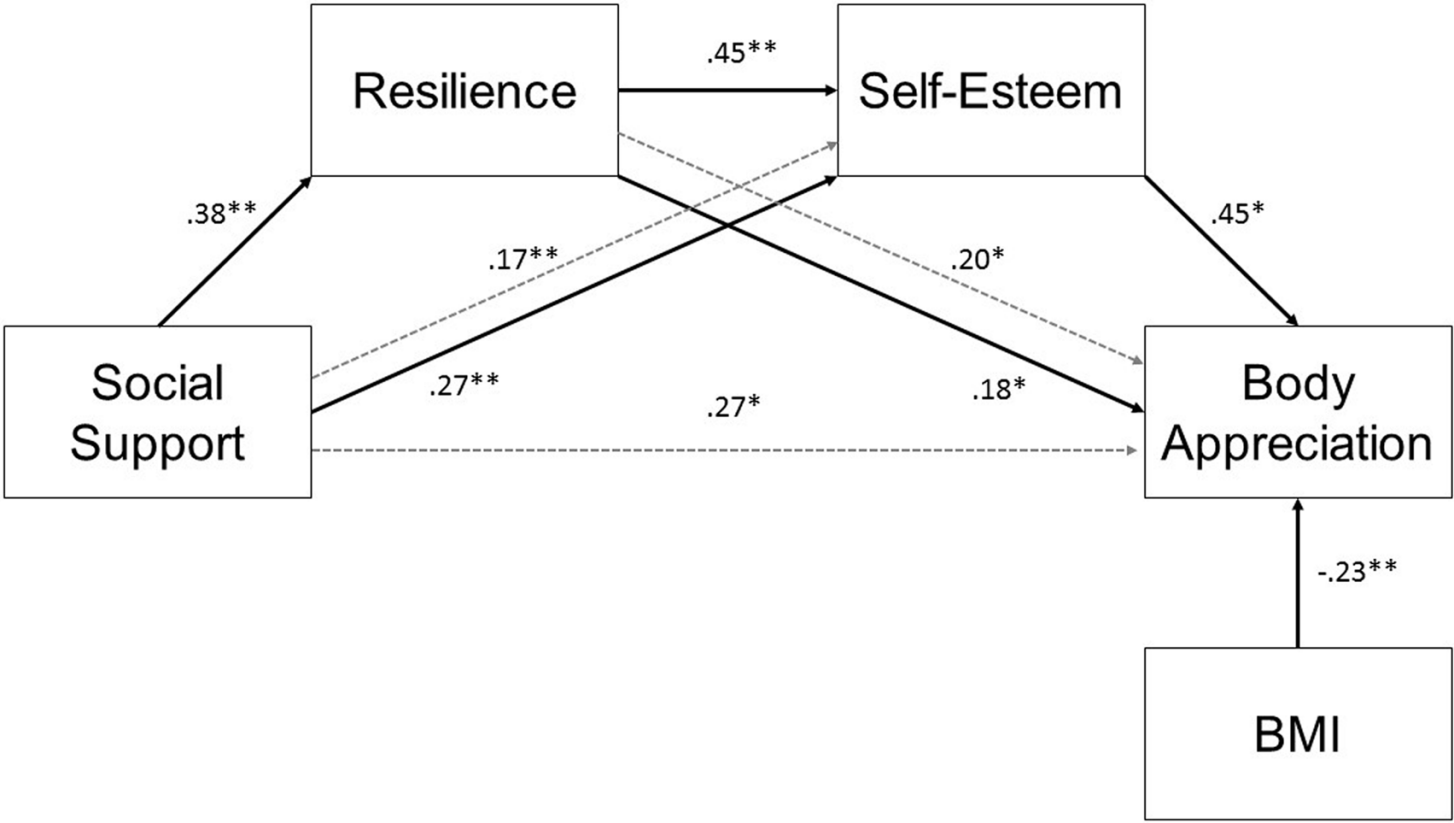
The final multiple mediational model with standardized regression coefficients. Indirect effects are represented by dashed arrows. *p<0.05; **p<0.01. BMI, body mass index.

When controlling for BMI, greater social support was related to greater resilience (*p*=0.001) and higher self-esteem (*p*=0.002). Similarly, greater resilience was related to both higher self-esteem (*p*=0.001) and body appreciation (*p*=0.017), and higher self-esteem was positively associated with body appreciation (*p*=0.001). All indirect effects were significant, including social support on self-esteem through resilience (*p*=0.001), resilience on body appreciation through self-esteem (*p*=0.001), and social support on body appreciation through resilience and self-esteem (*p*=0.011). That is, social support was not directly associated with body appreciation; rather, the effect was indirect, with greater social support being related to both greater resilience and higher self-esteem, which contributed to higher body appreciation in the sample. See Figure 2 for the final model presented with standardized regression coefficients.

#### Group differences exploratory analyses

We ran the final model separately for bisexual, lesbian, and queer women. See Table 2 for an overview of direct and indirect effects across groups. In bisexual women, the association between BMI and body appreciation (β=−0.340) was stronger than in lesbian (β=−0.140) and queer women (β=−0.226). Resilience was also more strongly related to body appreciation in the bisexual sample (β=0.345) than lesbian (β=−0.083) and queer (β=0.138) samples. Conversely, associations with self-esteem were generally weaker in bisexual women. For instance, resilience displayed stronger relations with self-esteem in lesbian (β=0.571) and queer women (β=0.445) than bisexual women (β=0.277). Similarly, self-esteem related more strongly to body appreciation in lesbian (β=.683) and queer women (β=0.579) than bisexual women (β=0.176). Finally, the multiple mediation effect was stronger in the lesbian (β=0.297) and queer samples (β=0.290) relative to the bisexual sample (β=0.166). The other notable difference that emerged was in lesbian women, where social support related more strongly to resilience (β=0.509) than in bisexual (β=0.292) and queer women (β=0.266).

## Discussion

This study examined a multiple mediational model of body appreciation in LBQ women. We hypothesized that social support would be indirectly related to body appreciation through resilience and self-esteem. We controlled for BMI, as it was significantly associated with body appreciation in our sample. Even when controlling for BMI, our hypothesized paths were significant. Consistent with previous research, perceived social support was related to both greater resilience and self-esteem overall.^32,35,36^ Thus, LBQ women in our sample who perceived they had positive support from family, friends, and others reported a greater ability to bounce back from stress and higher self-esteem. Resilience and self-esteem were also related in our sample, such that those reporting more resilience also reported higher self-esteem. In our model, both resilience and self-esteem were related to greater body appreciation. There is substantial evidence that self-esteem is related negatively to body dissatisfaction,^38,39^ and some evidence suggests it relates positively to body appreciation.^40^ Previous work has generally not considered the role of sexual identity. Thus, our study offers preliminary evidence that self-esteem is also associated with body appreciation in LBQ women.

As hypothesized, there was no significant direct association between perceived social support and body appreciation after controlling for resilience and self-esteem. Thus, perceived social support was related to body appreciation indirectly, through resilience and self-esteem, reflecting a full multiple mediation. In our sample, it appears that social support is a resource that aids LBQ women's successful adaption to stressful circumstances. Moreover, social support seems to be a source of self-esteem for this group, possibly through identification with a supportive and accepting community.^32^ Greater resilience was also related to self-esteem; previous research suggests that positive affect contributes to this association.^36^ Although not measured in this study, it is also plausible that resilience increases one's self-efficacy, thereby bolstering self-esteem. Of interest, both resilience and self-esteem were related to body appreciation in this group. It could be that perceived social support was a resource that strengthened resilience in our sample, helping participants cope with stressors such as discrimination, narrow societal beauty ideals, and weight stigma, which fostered self-worth and appreciation for their bodies.

Because research suggests body image differs across sexual identity,^18^ we ran the final model separately for bisexual, lesbian, and queer women as an exploratory analysis. However, these results should be interpreted with caution, given the small group sizes. Standard errors were larger than in the overall model, indicating lower reliability of parameter estimates, as would be expected with smaller samples. The most notable differences appeared between bisexual women relative to lesbian and queer women. For instance, BMI was more strongly associated with body appreciation in bisexual than lesbian or queer women. There is evidence that bisexual women are vulnerable to heightened body image concerns relative to lesbian women, in part because relationships with heterosexual men can encourage internalization of societal thinness ideals.^50^ Bisexual women in larger bodies might feel more pressure to conform to these ideals, and consequently have lower body appreciation.

Resilience and body appreciation displayed the strongest association in the bisexual sample. It is possible that bisexual women who feel they are successfully navigating the norms and pressures from both the heterosexual and gay communities experience a greater appreciation for their bodies. The direct effects of self-esteem on social support, resilience, and body appreciation were relatively weaker in bisexual women compared with the other groups, as was the indirect effect of resilience on body appreciation through self-esteem. It appears that self-esteem was less relevant to body appreciation for bisexual women in this study. Previous research has found that bisexual women have lower self-esteem than heterosexual and gay women.^51^ Although self-esteem did not significantly differ between groups in this study, it is possible the determinants of self-esteem in bisexual women are more complex.

Despite some of these intergroup differences, full multiple mediation was found in each group, providing support for the validity of the model. This indicates that social support is related to increased resilience and self-esteem, which contribute to greater body appreciation in LBQ women. Body appreciation offers promise as a protective factor against harmful media effects and low self-esteem.^21,23^ In addition, body dissatisfaction is a robust risk factor for disordered eating and EDs,^5^ which have considerable mental and physical health consequences.^52^ Thus, fostering positive body image in women who are both vulnerable to disordered eating^27^ and less likely to receive adequate treatment^28^ could be protective.

Results could be used both clinically and to inform future work. For instance, clinicians working with LBQ women could encourage their clients to build and strengthen their social networks, especially within the LBQ community.^30^ Clinicians might also help their clients process how they overcame difficult circumstances, which could foster a sense of resiliency. There are promising and brief interventions, such as an expressive writing program, with demonstrated efficacy to increase body appreciation^24^; however, these have not been tested or adapted specifically for LBQ women. Future work should assess whether existing interventions are appropriate for LBQ women, and consider incorporating themes of social support, resilience, and self-esteem to potentially improve outcomes for this group.

Positive body image research to date has not examined sexual identity. This is the first known study to examine factors related to body appreciation in LBQ women. Thus, this study is novel and offers preliminary evidence of factors that support positive body image in LBQ women. However, it has limitations. Self-report measures can result in biased and socially desirable responses. We assessed general resilience, rather than resilience specific to sexual orientation-related discrimination or stigma. However, resilience reflects both a response to adversity and positive adaptation to the daily challenges and stressors of life,^53^ which can buffer the effects of stress, particularly for those exposed to stigma/discrimination.^35^ Given the documented health disparities in LGBT individuals,^54,55^ it seems particularly important to identify protective factors, such as general resilience, self-esteem, and body appreciation, that could foster physical and mental health. Our sample was age diverse, and women identifying as queer were significantly younger than those identifying as lesbian. However, age was not significantly associated with, and groups did not significantly differ on, the outcome variables. It is possible that the small sample size precluded the ability to detect the nuanced effects of age in this study. As noted, group differences should be interpreted with caution given the small group sizes. However, because some paths appeared to differ between groups, future work should prioritize the recruitment of samples large enough to detect the unique contributors to body appreciation in each group. Data were collected before revision of the BAS. Nevertheless, the original measure displayed acceptable psychometrics in previous samples of women,^41^ and in this study. Finally, this study was cross-sectional. Future research should assess this model longitudinally, to investigate the temporal order of these relations.

## Conclusion

This study offers preliminary evidence that social support, resilience, and self-esteem help foster body appreciation in LBQ women. This is important, as body appreciation might be protective against mental health concerns and disordered eating, which are elevated in LBQ women. To date, the most empirically supported body image interventions are neutral regarding sexual identity; however, these results suggest LBQ women might benefit from interventions that consider sexual identity and target factors uniquely relevant for this group, such as social support, resilience, and self-esteem.

## Abbreviation Used

BAS: Body Appreciation Scale
BMI: body mass index
BRS: Brief Resilience Scale
LBQ: lesbian, bisexual, and queer
LGBTQ: lesbian, gay, bisexual, transgender, and queer
MSPSS: Multidimensional Scale of Perceived Social Support
RSES: Rosenberg Self-Esteem Scale
